# A Bird’s-Eye View on Polymer-Based Hydrogen Carriers for Mobile Applications

**DOI:** 10.3390/polym14214512

**Published:** 2022-10-25

**Authors:** Mohammadhossein Sharifian, Wolfgang Kern, Gisbert Riess

**Affiliations:** Montanuniversität Leoben, Chair in Chemistry of Polymeric Materials, Otto-Glöckel-Strasse 2, A-8700 Leoben, Austria

**Keywords:** hydrogen storage, polymers, solid-state hydrogen carrier, renewable energy, hydrogen mobility

## Abstract

Globally, reducing CO_2_ emissions is an urgent priority. The hydrogen economy is a system that offers long-term solutions for a secure energy future and the CO_2_ crisis. From hydrogen production to consumption, storing systems are the foundation of a viable hydrogen economy. Each step has been the topic of intense research for decades; however, the development of a viable, safe, and efficient strategy for the storage of hydrogen remains the most challenging one. Storing hydrogen in polymer-based carriers can realize a more compact and much safer approach that does not require high pressure and cryogenic temperature, with the potential to reach the targets determined by the United States Department of Energy. This review highlights an outline of the major polymeric material groups that are capable of storing and releasing hydrogen reversibly. According to the hydrogen storage results, there is no optimal hydrogen storage system for all stationary and automotive applications so far. Additionally, a comparison is made between different polymeric carriers and relevant solid-state hydrogen carriers to better understand the amount of hydrogen that can be stored and released realistically.

## 1. Introduction

In elemental form, hydrogen is not readily available on Earth; however, it can be naturally or industrially synthesized from its compound sources. A large percentage of the universe’s matter (approximately 75 wt%) is made up of hydrogen, making it one of the most abundant elements [1,2]. The combination of hydrogen with other elements makes it the tenth most abundant element in Earth’s crust. The gravitational force of Earth is insufficient to retain lightweight H_2_ molecules (unlike Jupiter and Saturn), and so elemental hydrogen (H_2_) unfortunately cannot be found in Earth’s atmosphere. It should be noted that hydrogen exists in three isotopes: protium, deuterium, and tritium, where protium with one proton and one electron is the main component of hydrogen.

**Figure 1:**
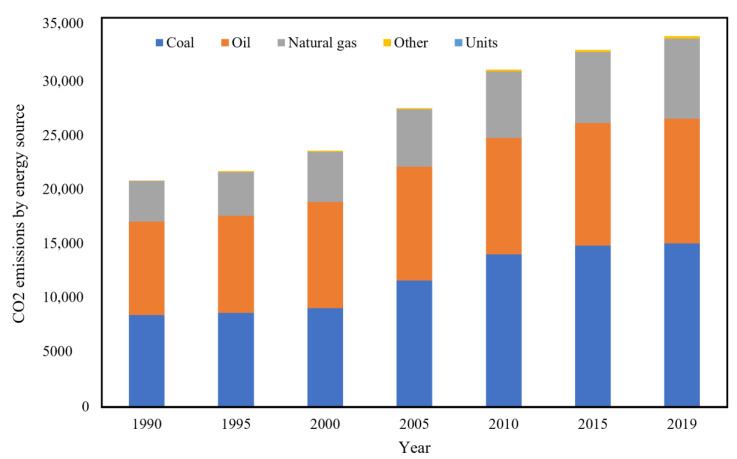
CO_2_ global emissions from various fuel combustion sources (IEA (2022) all rights reserved) [3].

As part of the net-zero emissions project, many countries aim to reduce greenhouse gas emissions by law. Because of the increasing population and number of industries, the emission of greenhouse gases such as CO_2_ is on the rise. Figure 1 demonstrates CO_2_ emissions from the most popular fuel sources since 1990. Globally growing CO_2_ emissions are a critical issue to be addressed in the near future. Net-zero projects suggest that CO_2_ emissions should be equal to the CO_2_ reduction processes or that clean energies should be used instead of carbon-based fuels [2,3].

The International Energy Agency (IEA) predicted that hydrogen-based fuels alongside other renewable sources would be one of the most important solutions for net-zero CO_2_ emissions by 2050. The political momentum for hydrogen use continues to grow every year. The advancement of hydrogen technologies and markets is crucial, since climate change remains the key impetus for widespread use of low-carbon hydrogen. Despite being the most abundant of all fuels, hydrogen has the highest energy content per unit mass [1,2,4]. These abilities mean that the governments of Canada, USA, Chile, Colombia, and Russia as well as European countries such as Germany, Spain, Netherlands, Norway, Portugal, France, Czech Republic, Hungary, and the United Kingdom have promulgated hydrogen strategies in their policies [5].

For commercial applications, H_2_ gas produced by various methods (such as water electrolysis, photonic, biological, nuclear, etc.) needs to be stored and transported to multiple locations, which is challenging and potentially dangerous due to H_2_ explosion risk [6]. Additionally, the use of hydrogen as a vehicle fuel requires the development of small tanks that can store large amounts of fuel [7]. In order to measure the amount of hydrogen gas in storage tanks, two instructions were proposed. Gravimetric capacity is defined as the weight of H_2_ stored per unit mass (expressed as kg_H2_ kg^−1^ or as a weight percentage (wt%)). If a storage tank weighs too much, then, since hydrogen has such a low molar mass, the amount of H_2_ stored in the carrier will be insufficient, and the cost of transportation will be too high. This is also a serious issue for mobile applications, as longer driving ranges together with much shorter refueling times should be a major advantage of hydrogen fuel compared to electric vehicles [8,9]. On the other hand, volumetric capacity determines tank volume. This parameter is defined as the amount of H_2_ absorbed per unit volume of absorbent, such as g_H2_L^−1^. In order to calculate the volumetric capacity of a material, it is necessary to know the material bulk volume or density. This parameter is mostly reported for the storage beds which adsorb H_2_ inside porous materials [8,9]. In the second step, further properties such as stability, durability, cyclability, and sorption kinetics of hydrogen carriers are also considered.

Honda, Hyundai, and Toyota manufacture and sell vehicles that use compressed hydrogen at 70 MPa as fuel, and so this is rapidly becoming the industry standard [10]. However, storing H_2_ at this pressure requires expensive cylindrical carbon fiber composite tanks which can handle highly compressed H_2_ gas without the risk of explosion. However, all new storage methods must meet or surpass the current benchmarks of compressed H_2_ storage technology in order to be viable. These storage tanks (70 MPa compressed H_2_, called type IV) mostly represent a gravimetric capacity of at least 5 wt% (the Toyota Mirai, for example, uses tanks that store 5.7 wt% H_2_ [10]) or volumetric capacity of approximately 40 g_H2_L^−1^. In order to achieve a higher gravimetric or volumetric capacity under mild conditions, various systems involving physisorption or chemisorption methods were reported [6,11,12,13]. The US Department of Energy (DOE) released ultimate targets for on-board hydrogen storage for lightweight vehicles. With a storage system cost of USD 0.226 per gram of hydrogen, the target of 6.5 wt% or 50 g_H2_L^−1^ must be reached. The operation and delivery temperature should range between −40 and +60 °C, while the maximum delivery pressure is considered 12 bar. It is vital that these systems be capable of delivering hydrogen that meets the quality standards needed for fuel cell vehicles [14].

**Figure 2:**
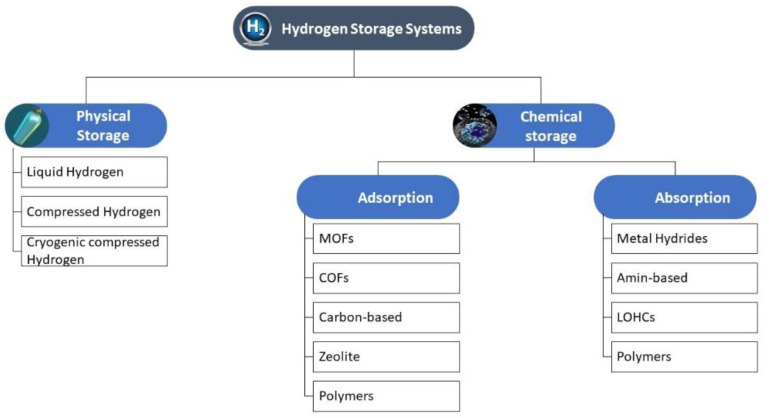
Hydrogen storage technologies.

All hydrogen carriers can be classified into two major groups: physically stored and chemically stored (Figure 2). The physical storage (PS) method refers to various methods based on the usage of compressed H_2_ gas, liquid hydrogen, and hydrogen storage in cryo-compressed conditions. PS is affected by changing storage conditions, such as pressure for compressed gas or temperature for liquid storage. It can also depend on both parameters in cryo-compressed storing systems. Despite its benefits, PS has some disadvantages, such as high energy consumption and special vessel requirements for high pressures without fuel leakage to avoid fires or bursts [7,15].

In contrast to PS, material-based or chemical storage (CS) relies on materials that can reversibly store hydrogen either as hydride or molecular hydrogen [7,15]. CS is predicted to have a higher hydrogen storage capacity at room temperature and lower pressures as well as a slower hydrogen release rate. There are two classes of chemical storage system, and each class has some subgroups based on the storage mechanism of absorption or adsorption (Figure 2). Metal hydrides such as LiBH_4_ or NaBH_4_ [16,17,18,19], amine-based materials [20], and liquid organic carriers (LOHs) [21,22] store hydrides through the absorption mechanism. In such systems, hydrogen is able to be absorbed and released repeatedly by using hydrolysis, ammonolysis, or thermolysis methods. On the other hand, the hydrogen carriers based on the adsorption mechanism are metal–organic frameworks (MOFs) [23], covalent organic frameworks (COFs) [24,25], and carbon-based carriers [26,27,28]. There are advantages and disadvantages to each family; however, the most important drawbacks are related to hydrogen capacity, hydrogenation/dehydrogenation kinetics, hydrogen purity, process conditions, and overall cost, since the recovery of hydrogen from these materials mostly involves energy-inefficient endothermic processes [2,7].

Up to now, a number of studies have begun to examine the use of polymers in chemical storage systems based on both mechanisms [29,30,31,32]. The main goal has been to find materials that can store hydrogen with a hydrogen capacity the same as or larger than the density of liquid H_2_. This review describes organic polymer materials which have been reported and studied as hydrogen carriers so far. One group is based on the adsorption of hydrogen molecules onto highly porous polymers, whereas the second involves absorption of hydrogen atoms through a reversible hydrogenation/dehydrogenation of active groups on polymer chains. Due to inherent advantages of polymers, such as low weight, easy production processes, higher safety, and easy handling, these systems are often recommended.

In the following, polymer-based hydrogen carriers characterized by both absorption and adsorption mechanisms are summarized. Additionally, organic frameworks and carbon-based materials are reviewed as organic solid-state hydrogen carriers in order to compare with polymeric systems.

## 2. Absorption-Based H_2_ Storage Systems

The chemical absorption of hydrogen in organic materials such as liquid organic hydrogen carriers (LOHCs), nitrides, borohydrides, and ammonia families has been variously studied for on-board use. These materials can offer higher gravimetric hydrogen uptake compared to metal-based hydrogen carriers that use adsorption mechanisms [4,7]. However, these groups are not totally irreversible and can be only used as either single- or dual-use fuels, where each use of these materials generates a waste that needs to be disposed of in environmentally critical processes. Furthermore, the purity of hydrogen released from polymers can be higher compared to small molecules. In the case of LOHC or other small molecules, during the dehydrogenation process, the purity of the released hydrogen could be decreased by the evaporation of small molecules. Due to the negligible vapor pressure of polymers, a risk of evaporation does not exist [33]. Other disadvantages include the instability of hydrogenated molecules as small molecules and the difficulty in controlling hydrogenation and dehydrogenation kinetics [34,35,36]. A certain number of hydrogenation/dehydrogenation cycles is needed to cover the ultimate DOE standard. Recently, hydrogen absorption through different polymers, such as polyketones, polyquinaldine, and intrinsically conductive polymers, has been studied in order to take advantage of small molecules and reduce their disadvantages.

### 2.1. H_2_ Storage in Functionalized Polymers

Despite the small molecules capable of storing hydrogen by chemical bonds, the lack of polymeric hydrogen carriers with the ability of chemical absorption was recognized in this area. For the first time, Nishide’s group studied the hydrogen uptake of polymers containing pendent organic groups which were investigated as LOHCs [37,38]. This idea was derived from Staudinger et al., who hydrogenated polystyrene during the decomposition of different polymeric materials [39]. At 473 K, polystyrene with various molecular weights was hydrogenated with a nickel catalyst. The results of relative viscosity and molecular weight changes were considered proof of successful hydrogenation. In the presence of catalysts, these polymers can store and release hydrogen through the forming and breaking of chemical bonds with hydrogen atoms.

A study by Kato et al. identified polyketones as promising materials for hydrogen carriers in which the reversible hydrogenation was studied by using electrochemical methods [37]. These polymers were hydrogenated in an aqueous solution versus the Ag/AgCl electrode at room temperature while dehydrogenation occurred at 80 °C. In this context, the hydrogenation of fluorenone polymers as a polyaromatic ketone was investigated in an aqueous solution in the presence of an Ir-based catalyst. ^1^H-NMR analysis of the polymer demonstrated that 94% of the H_2_ was released after 5 h with respect to the total weight of the sample. In addition, GC-MS was used to control the purity of the H_2_ gas evolved by the reaction. So far, the FTIR spectra of both small molecules and polymers show complete dehydrogenation as expected from their chemical formulas.

In another study by this group, a quinaldine-based polymer was reported as an alternative polymer for hydrogenation through the same method [38]. Due to the fact that the hydrogenation of quinaldine molecules requires a specific solvent and can only proceed at high temperatures, the use of this small molecule for real applications is highly energy-intensive [40]. Therefore, Kato et al. suggested quinaldine-substituted poly(acrylic acid) as a hydrogen storage carrier to achieve reversible hydrogen absorption of these molecules with the beneficial properties of polymers. Based on this study, it would be possible to prepare electrochemical engines using reversible hydrogen carriers operating under mild conditions. Water is used as the source of hydrogen, while the polymer-coated carbon electrode is considered as the cathode vs. the silver anode. The Ir-based catalyst is used to accelerate the hydrogenation reaction of the polymer in the aqueous solution, and the dehydrogenation is also carried out by simply heating the samples in water. The yield of hydrogenation in the best performance was reported to be 92%. Recently, these authors continued this field by proposing poly(6-vinyl-2,3-dimethyl-1,2,3,4-tetrahydroquinoxaline) (4H-PVQ) gel instead of a coated polymer for reversible hydrogenation under the same mild conditions [41]. The prepared polymer was hydrogenated at 60 °C and ambient hydrogen pressure and was dehydrogenated over 5 h under 120 °C and air. However, this system does not require an electrochemical cell for hydrogenation and dehydrogenation, and the source of the H_2_ is not water. Both the hydrogen absorption and release procedures were conducted in the presence of the same iridium complex as a catalyst. An increase in the number of nitrogen elements in the aromatic parts could increase the absorption capacity of such polymers [42]. The calculated H_2_ uptake in this polymer was up to 2.6 wt% (related to the repeating unit), which is a fairly high number for a chemical solid-state hydrogen carrier so far. This group published another article to improve the hydrogenation rate of quinoxaline-based polymers [43]. A poly(vinyl diphenylquinoxaline) was obtained by modifying bromo-quinoxaline to give 6-vinyl-2,3-diphenylquinoxline and polymerizing it in THF. The sample was swollen with N-methylpyrrolidone and warmed at 180 °C in the presence of the Ir-based catalyst in order to undergo hydrogenation. According to ^1^H NMR analysis, 100% dehydrogenation occurred only after 60 min at 180 °C, which is faster than PVQ. It should be noted that, in addition to increasing the rate of dehydrogenation, this modification also reduced the maximum mass absorption from 2.53 wt% H_2_ for PVQ to 1.28 wt% H_2_ for poly(vinyl diphenylquinoxaline).

Kato et al. studied the effects of an iridium catalyst on poly(vinylfluorenone) hydrogenation under atmospheric pressure by forming chemical bonds [44]. A higher amount of fluorenone groups on the polymeric chains demonstrated a higher hydrogen density and storage capacity. FTIR analysis showed an approximately total exchange of ketone groups to alcohol groups in the electrochemical cell bath at 100 °C after 5 h. Nevertheless, the maximum theoretical amount of H_2_ absorbed per fluorenone group is only 1 wt%, and it cannot exceed that level in real experiments. This means that a 100% hydrogenation yield could only absorb 1 wt% of hydrogen gravimetrically, which is significantly less than the DOE standard for mobile applications. Despite the fact that this polymer is an acceptable material for rechargeable proton-exchange membrane fuel cell systems, more research on poly(vinylfluorenone) is worthwhile [45]. The results of this study were followed by another analysis on the hydrogenation of poly(methyl vinyl ketone) in the presence of an iridium catalyst [42,46]. Poly(methyl vinyl ketone) can theoretically absorb and evolve a higher amount of hydrogen in comparison to fluorenone. Dehydrogenation of poly(3-buten-2-ol) at 180 °C in 1.5 h resulted in a hydrogen density of 2.8 wt%. The poly(3-buten-2-ol) had a lower activation energy of dehydrogenation than the fluorenol polymer (37.0 kJ/mol). However, the standard reaction enthalpy for dehydrogenation of this polymer was larger than that of the polyfluorenol (ΔH^o^ = +54.9 kJ/mol) [47]. Recently, acetone-substituted poly(allylamine) was investigated as a hydrophilic solid-state hydrogen carrier with a maximum hydrogen storage capacity of 1.5 wt%. Hydrogenation was carried out in the presence of the Ir-based catalyst under ambient conditions. An aqueous solution of sodium borohydride (NaBH_4_) was used for in situ hydrogenation to obtain an isopropanol-substituted poly(allylamine) derivative. Despite the lower hydrogen storage mass, acetone-substituted poly(allylamine) could provide a non-explosive fuel system that could be hydrogenated through a safe procedure. However, the complete dehydrogenation with 100% yield occurred only after 8 h at 95 °C, which is slower than other carrier groups [48]. This study, as well as similar studies that have used the same method, is extremely useful for future research, due to the mild conditions of hydrogenation and dehydrogenation (*T* < 150 °C and *p* < 3 bar) as well as the high reversibility. The maximum storage capacity for the studied polymers is, however, far below the DOE’s requirements, as shown in Table 1. Therefore, polymers synthesized from hydrogen carriers with higher hydrogen storage capacities (suggested in the last three rows) may provide a realistic solution for hydrogen carriers for mobile applications [22,49]. A study on the hydrogen storage ability of these selected polymers is being conducted by our group. Since polymers are categorized as solid-state carriers, the conditions of hydrogenation/dehydrogenation might be adopted in comparison to small molecules. It should be noted that the hydrogenation procedure of these polymers can occur in safe places, while the dehydrogenation condition could meet the DOE standard. Hydrogenation of solid-state hydrogen carriers would be possible under severe conditions (i.e., high pressure and high temperature) in this case. Nevertheless, new studies are focusing on the moderation of hydrogenation conditions by using different catalysts or by benefiting from new functional groups. Dehydrogenation, however, can be performed under conditions suggested by DOE targets.

In addition to being affordable, lightweight, non-explosive, non-volatile, and manufacturable, these polymers have additional advantages such as the ability to be mixed with other systems. In order to obtain a better understanding of chemical hydrogenation/dehydrogenation of polymers through the absorption mechanism, studies need to be conducted with a focus on hydrogen capacity under various conditions for mobile applications. Additionally, the rechargeability and the purity of released hydrogen are some other important parameters that need to be to studied in the future. Moreover, the new materials must be tested in order to enhance the kinetics and thermodynamics of H_2_ uptake.

### 2.2. H_2_ Storage in Intrinsically Conductive Polymers (ICP)

**Figure 3:**
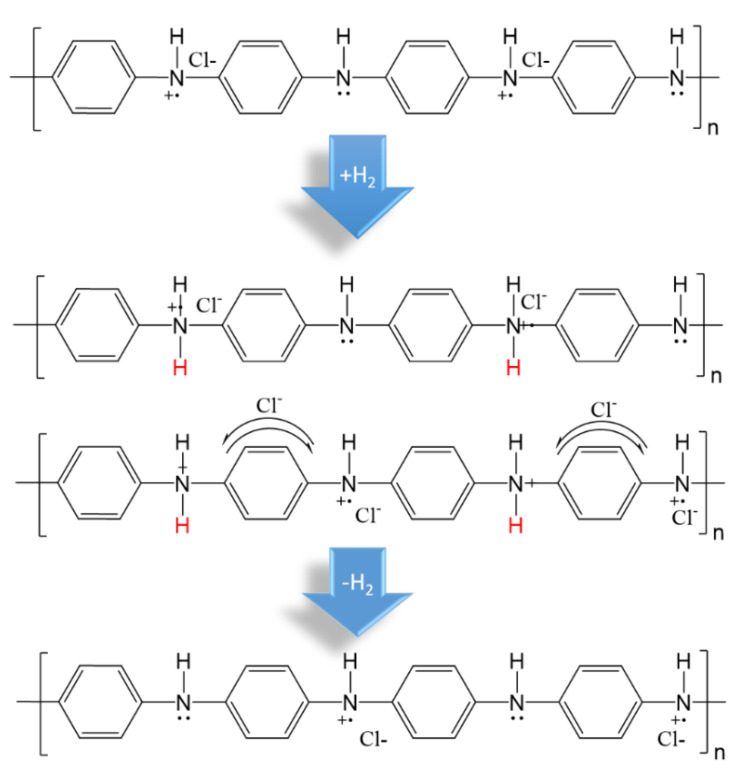
Mechanism of hydrogen storage in polyaniline [11].

Intrinsically conductive polymers are a class of exciting organic materials that conduct electricity due to the presence of a metal atom in the center of the chains or because of unpaired electrons or π-electrons at each sp^2^ hybridized carbon atom. These polymers, which are sometimes termed synthetic metals, can be doped to exhibit electrical conductivity close to that of metals. Over the last three decades, the potential of ICPs has been investigated in various applications. Despite the electronic applications which are well known for these polymers, some ICPs have been proposed for coatings, skin tissue engineering, wound care, biosensors, and energy and gas storage [11,53,54,55]. Among ICPs for hydrogen storage systems, polyaniline (PANI), polypyrrole, and polythiophene have been investigated due to their excellent thermal, electrical, and environmental properties. The high hydrogen absorption capacity is not only related to the high porosity of crosslinked systems, but it can be enhanced by increasing the number of conductive groups which interact with the hydrogen atoms (Figure 3) [11]. Polyaniline, with the highest thermal stability and acid/base reactivity, exhibited a hydrogen sorption capacity of 6 and 8 wt% at ambient temperature and 8 bar, as reported by Cho et al. [56]. Besides the fact that these results were not generally corroborated by other studies on the H_2_ uptake of polyaniline, the gravimetric hydrogen capacity was also significantly decreased in the second and third cycles of hydrogen absorption [57].

A number of PANI materials with various morphologies and nanostructures have been studied using the notion that PANI materials possess exceptional electronic properties and that the delocalization of charge along the polymer backbone might generate a large number of hydrogen-binding active sites [58,59,60]. The PANI morphologies are very diverse, but fibers and nanofibers have attracted the most attention, since they are one-dimensional and can be easily prepared in controlled diameters [57]. Increasing the conductivity of PANI by doping it with protonic acids results in the creation of cellular textures and a microstructure with nanosized pores (30 nm) that can adsorb small molecules such as hydrogen. Chen et al. reported activated rectangular polyaniline-based carbon tubes with high specific surface areas ranging from 1680 to 2415 m^2^ g^−1^ [61]. Different mass ratios of polyaniline/carbon nanospheres in the composition resulted in a variation in hydrogen uptake, the highest reported number being 5.2 wt% at 77 K and 5 MPa. However, only 0.62 wt% H_2_ was stored in the same structure at room temperature, even at higher pressure. Sevilla et al. reported almost similar composites produced from micro-mesoporous carbon (derived from polythiophene) and with activated polypyrrole, with a H_2_ uptake of 7.3 wt% at 77 K and 2 MPa [62].

Some PANI types have recently been alloyed with metal composites to improve their electrochemical and hydrogen storage properties [32,63,64,65]. These studies illustrated that charge transfer and synergistic hydrogen transmission were enhanced by combining inorganic complexes with organic polymers. However, the hydrogen storage capacity of these alloys was not studied. Recently, Mahato et al. reviewed and summarized ICPs reported for hydrogen storage and fuel cell applications. The molecular structure, synthesis, mechanism of hydrogen storage, and effective parameter in hydrogen storage were briefly discussed. It has been demonstrated that the hydrogen uptake of ICP structures depends on the unique electronic properties, surface area, and porous structure of the polymers [11].

## 3. Adsorption-Based Storage Systems

As noted before, hydrogen can be stored in nanoporous materials through an adsorption mechanism, where H_2_ molecules are mainly adsorbed to the inner surface of a material. In order to explain these phenomena, we need to understand that hydrogen molecules can only interact with the surface of solid materials through van der Waals forces as well as electrostatic and orbital interactions [2,66]. These interactions are usually quite weak, since H_2_ does not have any charge, dipole moment (only a small quadrupole moment), or high polarizability [66]. As a result, the large amount of hydrogen adsorbed onto porous materials under cryogenic conditions decreases as temperature increases. Significant amounts of H_2_ can adsorb onto nanoporous materials at any pressure and at low temperatures such as 77 K. The same pattern was also observed with increasing pressure at a particular temperature [67]. A general trend has been observed for nanoporous materials, described as the correlation between an increase in gravimetric capacity and an enhancement in the specific surface area (known as Chahine’s rule). Therefore, increasing the specific surface area of nanoporous materials (expressed gravimetrically in m^2^ g^−1^) should lead to larger gravimetric H_2_ uptake in the absence of physical limits [68]. The new studies confirm that the correlation between the specific surface area and the gravimetric capacity of porous materials is roughly linear, but the correlation of that with volumetric capacity is more complicated [13].

As described before, the gravimetric capacity determines the weight of a storage tank, while volumetric capacity determines the volume of that container. It is noticeable that the gravimetric capacity of most existing hydrogen carriers is still not suitable for mobile applications. Nevertheless, it is possible that polymers constructed from light elements with the appropriate pore size could have a practical gravimetric capacity at ambient temperatures and pressure. The purity of the hydrogen source is important to ensure that the hydrogen delivered from the nanoporous materials (as storage system) is also pure. These impurities can preferentially decrease the real storage capacity of carriers. Impurities can also contaminate the materials’ pores, thus leading to a loss in hydrogen capacity and reproducibility [14].

The intrinsic microporous polymers, conductive polymers, hyper-crosslinked polymers, and COFs are members of the groups of nanoporous polymers which are recommended for hydrogen storage, based on the mechanism which has been reported for MOFs and carbon-based materials [28,31].

### 3.1. H_2_ Storage in Polymers of Intrinsic Microporosity (PIM)

**Figure 4:**
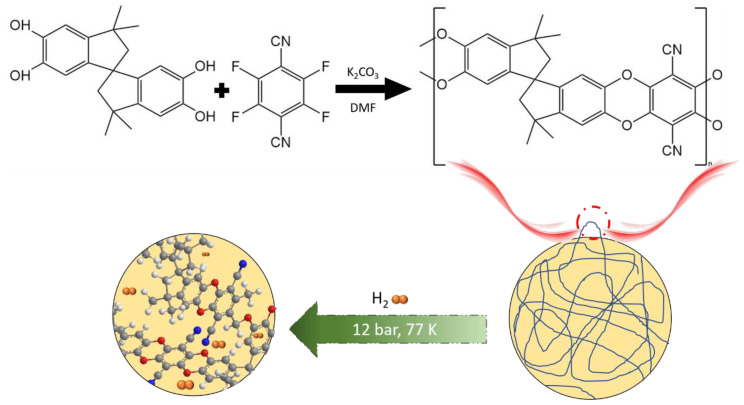
Schematic representation of PIM-1’s repeating linked units containing the H_2_ molecule.

PIMs are amorphous polymers that do not require a network of covalent bonds in order to demonstrate microporosity [69]. These polymers are defined as “a continuous network of interconnected intermolecular voids, which forms as a direct consequence of shape and rigidity of proposed synthesized polymers” [70]. As membranes, PIMs are built of highly inflexible and twisted molecular frameworks which contain an abundant number of interconnected micropores (pore size < 2 nm) and ultra-micropores (pore size < 0.7 nm) [69]. A number of well-known classes of amorphous polymers provide intrinsic microporosity, as demonstrated by gas permeability (e.g., polyacetylenes [71], fluorinated polymers [72], polynorbornene [73], and polyimides [74]). These properties are being exploited in the field of polymer membranes, where they are described as "ultra-permeable" polymers [69]. At first, these membranes were used for water treatment and organic material separation; however, with the introduction of PIMs to gas separation, they were also evaluated as hydrogen carriers [69]. It should be mentioned that the PIMs produced through condensation polymerization of porous aromatic alcohol with fluorinated aromatic structure can provide lightweight porous frameworks containing various active elements (Figure 4). These systems maintain porosity even under wet conditions, which is an advantage over MOFs and COFs [75].

Ramimoghadam et al. reviewed most of the articles regarding the hydrogen storage of PIMs [75]. The PIMs were able to store a maximum of 4% of gravimetric hydrogen at 77 K and 10 bar even in the presence of a catalyst, according to this review. Due to the lack of research on the hydrogen storage capacity of PIMs at room temperature, the hydrogen stored in a PIM is not high enough to meet DOE requirements. The challenge with PIMs is their low surface area, which can be overcome by using modified monomers or specific moieties/subunits for synthesis or post-processing treatments of fabricated PIMs [31]. Ramimoghadam et al. reported the effect of thermal treatment of PIM-1 in order to improve the hydrogen storage capacity. With a surface area between 443 and 744 m^2^ g^−1^, treated PIM-1 adsorbed 4.6 wt% of H_2_ at 77 K and 100 bar [76]. The study of PIMs for higher hydrogen capacity at cryogenic temperatures continues, but more research is needed to examine the storage of hydrogen by PIMs at room temperature. With respect to practical hydrogen storage, greater attention must be paid to measuring and reporting the reversible uptake rather than the total uptake.

A PIM-based composite has been reported by Bambalaza et al. using the blending of a metal–organic framework made of zirconium and PIM-1 [77]. The final composites contain micro-mesoporous pore size distributions, considered to be useful materials for improving hydrogen uptake. In comparison to solitary MOF, hydrogen storage analysis of PIM/MOF composites shows an improvement of approximately 30%, both gravimetrically and volumetrically. By using this method, which is widely studied in the field of PIM-based membranes, the final material achieves a much higher BET surface area as well as a higher pore volume compared to PIMs.

### 3.2. H_2_ Storage by Clathrates

**Figure 5:**
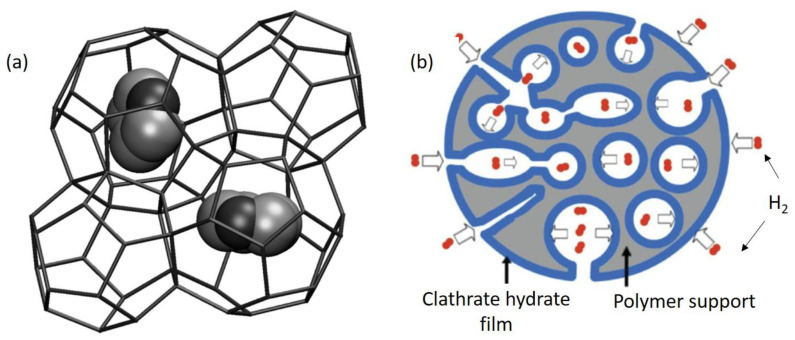
(**a**) Schematic clathrate structure, trapping a guest in a small cavity (<9A); (**b**) schematic illustration of clathrate hydrate dispersed on a polymer support (copyright Wiley Online Library 2008) [83].

During the 1810s, Sir Humphrey Davy recognized that an aqueous solution of clathrate turns into a solid when it is cooled below 9.0 °C [78]. After that, researchers have reported more than 100 similar compositions with the same behavior over the last century. Davidson applied the term “gas hydrate” to these solids, but the appropriate designation for these compounds is clathrate hydrate [79]. Clathrate hydrates, by definition, are compounds in which guest gas molecules are trapped inside host water molecules (Figure 5a). The gas is only held inside the cavities by physical bonds of the water cages, and no chemical reactions are involved in the storing process. Over several decades, gas hydrate or clathrate hydrate research has evolved from being an academic interest to being an alternative to the oil and gas industry for “flow assurance” [80,81]. Several advantages are offered by clathrate hydrates as a hydrogen storage material. Firstly, the storage material is pure water, which means that when the hydrogen from the hydrate is emitted, the only byproduct is pure water, which is reusable and compatible with hydrogen fuel cells. Secondly, the formation and decomposition kinetics can be very fast; thereby, completely converted hydrogen hydrate can form in minutes from powdered ice and in h from a solid chunk of ice. The combination of all of these factors, along with the abundance of water, could provide a cheap hydrogen storage method. It could be argued that the major disadvantage of clathrate hydrates is their high formation pressure [80,82,83].

There have been some articles reporting the preparation of clathrate hydrates and semi-clathrate hydrates on the surface of various polymers to provide gas storage systems (Figure 5b) [84,85]. Su et al. reported a supported hydrogel for the storage of H_2_ and compared it to a simple clathrate. The polymer hydrogels result in a faster and more efficient formation of hydrogen clathrates, suggesting faster gas storage kinetics in clathrate-based technologies [83]. Over the past two decades, a few reviews summarized articles dealing with this subject [80,86,87]. However, this field is still in development and will require careful studies of appropriate materials and conditions.

Moreover, hydrogen-release experiments require a better understanding of these systems’ abilities. In addition, machine learning methods could provide trustworthy information in a short time frame [80]. The next phase of research could concentrate on improving the synthesis conditions, reducing production pressure, and also studying the hydrogen desorption conditions simultaneously.

### 3.3. H_2_ Storage in Covalent Organic Frameworks (COFs)

COFs contain an anatomically precise spatial assembly of molecular organic building blocks, which offers a great deal of design space. These crystalline polymers are built from organic linkers via reversible covalent bonds, leading to the formation of porous networks. The various sizes, symmetries, and connectivities of the COF linkers lead to the specific geometry, thereby having attracted much interest in recent years [24,88,89,90]. Nanoscale channels and spaces formed by the 2D and 3D COF scaffolds are ideal environments for molecule storage and filtration [89,91]. Moreover, COFs are advantageous for applications that rely on charge carrier transport, including optoelectronics and electrochemical energy storage, due to their regularity and connectivity. All of these factors make COFs a good candidate for solid hydrogen storage [25,89]. Kalidindi et al. provided a detailed review of H_2_ storage by COFs [24]. Moreover, a review recently summarized the new articles in this field related to 2D and 3D COFs [90]. In addition to the surface area and the pore volume of COFs (which are the effective parameters for gas adsorption), the functional groups are an important parameter in the adsorption of H_2_ molecules, while, at the same time, they can improve storage ability and control the release rate because of the absorption mechanism [24].

**Figure 6:**
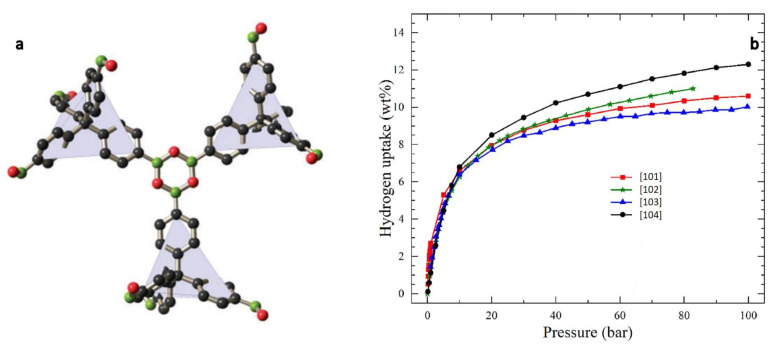
(**a**) Chemical structures of classic boron-containing COF-102 (copyright Royal Society of Chemistry 2011) [95]; (**b**) Comparison of four simulated absolute hydrogen uptake capacity of COF-102 at 77 K reported. (Copyright Elsevier 2022) [99].

High production costs, environmentally unfriendly procedures, and higher selectivity to CO_2_ in comparison to H_2_ are some important drawbacks of COFs when they are considered as hydrogen storage carriers for mobile applications. Furthermore, COFs, like other porous materials, require high pressure and cryogenic conditions to improve their storage capacity, and many articles have shown that room conditions reduced their wt% H_2_ uptake [25,90]. Even doping the COFs with active metals such as Li does not achieve the required standard in ambient conditions [92]. An article published recently suggested a hydrogen carrier based on scandium atoms modified with COF-1 [93]. The reversible hydrogen uptake of this composite was estimated to be 5.23 wt% at 300 K based on molecular dynamics in the CASTEP simulation. The DOE standard would be met if the experimental results could confirm the computational ones. Tong et al. simulated the volumetric hydrogen capacity of various COFs using a database of 449 synthesized COFs [94]. At 77 K and 80 bar, COF-102 showed the highest volumetric hydrogen adsorption of 56.1 g L^−1^, which might be explained by the structure of COF-102. Thus, tetrahedral units connected around the B_3_O_3_ ring in a triangular fashion provide high electron density that can lead to better interaction with H_2_ molecules (Figure 6a) [95]. The theoretical calculation and experimental measurement of different studies agreed on a more than 10 wt% hydrogen uptake for COF-12 at 77 ^o^C (Figure 6b) [96,97,98,99]. An investigation of the capacity of COFs to store H_2_ at ambient temperature and pressure is missing in this article. In spite of this, there is still a wide gap between academic studies of COF hydrogen carriers and useful commercial products.

## 4. H_2_ Storage in Metal–Organic Frameworks (MOFs)

**Figure 7:**
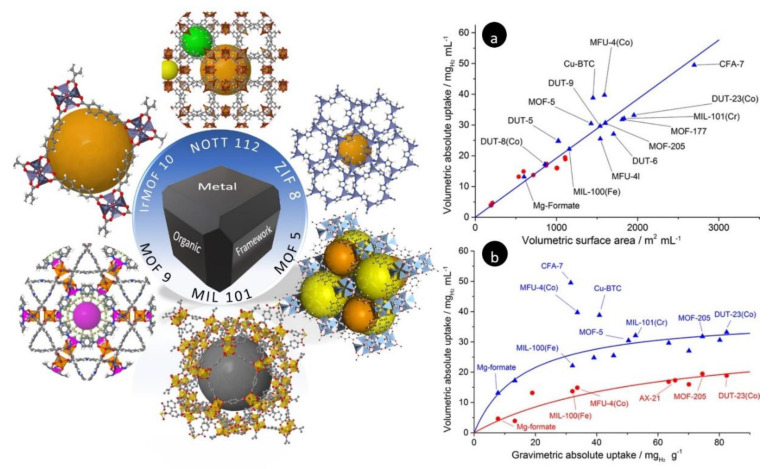
(**Left**) Schematic representation of some MOFs with high gas storage properties (copyright https://www.chemtube3d.com) (accessed on 14 April 2022); (**a**) linear relation between the volumetric hydrogen uptake at 77 K and 2.0–2.5 MPa and the volumetric surface area; (**b**) volumetric vs. gravimetric hydrogen uptake of various MOFs at 77 K and 2.0–2.5 MPa. The volumetric uptake was calculated using packing density (red) and single-crystal density (blue) [107].

A MOF is a highly porous material which can be synthesized using solvothermal reactions of metals with organic materials in organic solvents, particularly those that have high boiling points, such as dimethylformamide [100,101]. MOFs are built from three main parts: inorganic metal centers, organic ligands, and the topology of a framework. Each part can contribute different physical and chemical properties to the highly porous structure of MOFs. There have been more than 1000 MOFs synthesized and reported in the past half-century that have different pore sizes and pore volumes which have been realized by changing the structure of these three parts [102,103,104]. Figure 7 illustrates the structure of some of the most important MOFs studied for H_2_ storage.

Regarding H_2_ gas sorption, the metal part of MOFs can interact with the hydrogen gas molecules, while the interaction can be easily suspended by heating and/or evacuation without damaging the framework structure. The H_2_ molecules can bind to the open metal sites (unsaturated metal ions in the framework of MOFs) more strongly than with physisorption inside pores. Therefore, non-similar to nanoporous carbon composites, MOFs with higher numbers of open metal sites can lead to better H_2_ uptake [13]. One of the most reported carriers is the MOF topology similar to zeolite, called ZIF [105]. Several ZIFs exhibited promising results for storing H_2_ gas. For example, ZIF-8 stored 3.3 wt% H_2_ at 77 K and 30 bar, which is the highest amount for ZIFs [106]. So far, the highest gravimetric percentage of H_2_ reported for MOFs is 8.1 at 77 K and 90 bar for a zinc-based framework [101]. As with other solid-state hydrogen carriers, the storage capacity of MOFs is extremely dependent on temperature and pressure; at 298 K and 90 bar, the same zinc-based MOF showed only 0.5 wt% H_2_ uptake [101].

Several articles suggest that improving the surface area and pore size increases the hydrogen uptake of MOFs. Balderas-Xicohténcatl et al. compared the hydrogen storage capacity of MOFs with the specific surface area (Figure 7a). It was found that the volumetric hydrogen uptake was linearly related to the volume of specific surface area. Furthermore, a logarithmic relation between the volumetric and gravimetric H_2_ uptake of the MOFs was demonstrated (Figure 7b). Moreover, the interpenetrated MOFs had higher hydrogen capacities than other MOFs, and one interpenetrated MOF (CFA-7) displayed the highest volumetric hydrogen storage capacity at 77 K and 2.0 MPa [107].

Despite the good results at 77 K, the hydrogen absorption at room temperature is still not sufficient, due to the low enthalpy of hydrogenation even under high pressure. With a surface area of 6411 m^2^ g^−1^ at 77 K and 80 bar, the Zn_4_O(bpdc)(btctb)_4/3_ (DUT-32) MOF, prepared with ditopic (bpdc-4,4′-biphenylendicarboxylic acid) and tritopic (btctb-4,4′,4′-[benzene-1,3,5-triyltris(carbonylimino)] trisbenzoate) linkers, adsorbed 14.21 wt% H_2_. The absorption of this MOF was not measured under room conditions; nevertheless, the storage capacity was significantly decreased upon reducing the pressure of the initial H_2_ gas (from 14.21 wt% at 80 bar to 7.8 wt% at 53 bar) [108].

There are several methods suggested to improve H_2_ absorption through MOFs, such as doping with metals or catalysts [109,110], providing metal centers for MOFs [111,112], or combining these methods [113,114]. Despite the fact that each method can improve some properties of MOFs, a promising system for hydrogen storage at room temperature has not been achieved yet. Some reviews summarize the hydrogen storage data reported in various MOFs and MOF composites [23,31].

## 5. H_2_ Storage in Carbon-Based Hydrogen Carriers

Among various solid-state hydrogen carriers, porous carbon-based materials are the most frequently investigated for reversible H_2_ storage due to their high surface area, low density, high thermal and chemical stability, and ease of manufacturing at low cost. Carbon aerogels, carbon nanotubes, carbon fibers, and graphene are members of the carbon family which have been widely studied as hydrogen carriers [28,115]. It should be noted that both natural and synthetic nanoporous carbon materials could be used for large hydrogen storage.

Hydrogen storage in carbon materials has been investigated since the first study of H_2_ adsorption in active carbon in 1926 [116]. According to Schimmel et al., H_2_ interaction with carbon-based materials relies on weak van der Waals forces, and these low adsorption forces ruled out any possibility in which hydrogen might adsorb into the narrow channels between the carbon structures. This means that carbon materials can only adsorb hydrogen molecules onto small pores near the surface, and a higher surface area leads to greater storage capacity, as in the case of porous materials [117]. However, the gravimetric storage capacity of these materials does not correlate linearly with the specific surface area at larger surface areas [13]. As a result, the capacities of carbon materials analyzed for H_2_ uptake tend to correlate linearly with their surface areas and nanopore volumes [115].

Panella et al. [118] reported a correlation between hydrogen storage capacity and surface area for various commercial carbons under room temperature and cryogenic conditions. Enhancement of carbon-based surface area from 22 to 2564 m^2^ g^−1^ resulted in a linear increase in hydrogen uptake from 0 to more than 0.5% and from 0 to more than 4% at 298 and 77 K, respectively. Similar behavior was observed for the correlation between storage capacity and pore volume size. Furthermore, the studies demonstrated that CO_2_ adsorption analysis (instead of traditional N_2_ adsorption) provided a better correlation between hydrogen storage capacity and micropore size, reported for activated carbons with surface areas ranging from 890 to 3000 m^2^ g^−1^ [119]. Consequently, modified carbon materials with a higher surface area were investigated for obtaining adequate H_2_ uptake [62,120].

**Figure 8:**
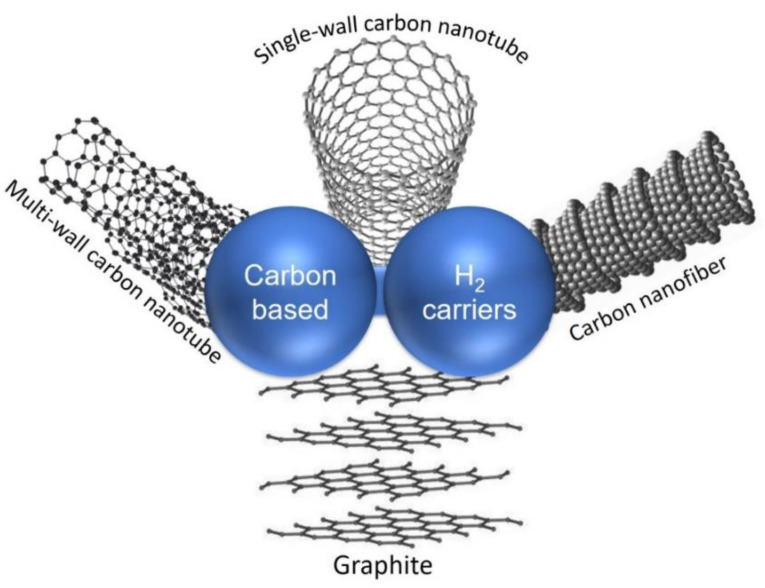
Most investigated carbon-based hydrogen carriers.

Since the surface area cannot infinitely increase, another important method to improve storage capacity is functionalization of the carbon-based carriers. In this regard, the carbon materials were doped with lightweight metallic elements which were able to increase the polarity of carbon composition for higher H_2_ adsorption [117,121]. Functionalizing the carbon materials in order to adjust pore size and volume also plays an important role in the H_2_ storage systems. According to Masik et al. [122], hydrogen storage capacity in porous carbon decreases linearly with increasing pore size from 1.2 to 2.3 nm and 3.1 nm. In this way, decreasing the pore size from 3.1 to 1.2 nm improved the hydrogen storage of the carbon materials (with the same specific surface area) by 70%. In addition, there is no general agreement on the optimal pore size; it is believed that the adsorption enthalpies, as well as pore density (pore space divided by the total volume), will decrease as the pores become larger [12]. So far, dramatic advances in hydrogen storage for numerous carbon materials, such as activated carbons, graphite, carbon nanotubes, carbon fibers, and templated porous carbons, are well documented (Figure 8).

### 5.1. Activated Carbon (AC) Hydrogen Carriers

Generally, ACs are generated from chemical and physical activation of wood, coal, and other natural sources, which leads to a low mass density and high surface area. The hydrogen storage ability of ACs was studied firstly by Chahine and Bose in the 1980s, using the AX21 activated carbon [68,115]. The final stored amount of hydrogen reported in this article was 2 wt% at ambient temperature. After that, many studies were focused on activated carbon modification in order to achieve higher specific surface area as well as adsorption capacity. In this regard, Zhao et al. reported 6.6 wt% adsorption capacity at 77 K with a surface area of 3451 m^2^ g^−1^ after treating various ACs with KOH [123]. However, AC studies highlighted the limited amount of adsorption sites on the surface of modified ACs under ambient conditions, even at high specific surface area [124]. Furthermore, the hybridization of ACs with different metals, such as palladium or platinum, was unable to improve the H_2_ uptake of the ACs. The hydrogen storage capacity of platinum-doped AC/metal–organic framework hybrid composites at 298 K and 100 bar was studied by Lee and Park. They adsorbed 2.3 wt% H_2_ under this condition, which is significantly larger than that of raw ACs [125]. The palladium nanoparticle-doped ACs also showed a maximum uptake of 1 wt% H_2_ at 10 bar and 298 K. Zhao et al. demonstrated that increasing the Pd amount could also have a negative effect on the final adsorption capacity, even under cryogenic conditions [126]. However, none of these results could yet meet the DOE requirement for mobile applications. The activated carbons still suffer from a lack of adsorption capacity under ambient conditions.

### 5.2. Graphite-Based Hydrogen Carriers

Graphite, with its sp^2^ hybridized sheet-like structure, is one of the most abundant forms of carbon in nature wherein carbon atoms are bonded together by van der Waals forces, resulting in parallel-oriented sheets (Figure 8). This structure provides a high surface area with suitable porosity to adsorb and trap small molecules. A variety of methods for synthesizing graphite and modifying its structure have been described in view of its various applications. Graphene, graphite nanoplatelets, graphite intercalation compounds, graphene oxides, and expanded graphite are some of the important members of the graphite family [124,127].

The improvement in storage ability has been part of energy studies since 1971, when the storing of hydrogen in potassium graphite was published by Waranabe et al. [128]. Due to the relatively high interaction energy between hydrogen molecules and graphite carbon atoms, the weight of hydrogen adsorbed to non-modified graphite is out of consideration for commercial applications. Furthermore, Ahluwalia et al. modeled expanded neutral graphite as a suitable hydrogen storage carrier for automotive fuel applications. In that study, 5.5 wt% and 40 g L^−1^ of hydrogen gas was captured between the modified graphene layers under cryogenic conditions [129]. Graphene oxides and their alkali metal hybrids increased the hydrogen storage capacity of these carriers by increasing the interlayer spacing of graphite sheets [127,129,130]. Nevertheless, even reduced graphene oxide hybridized with various active metals has been unable to meet the DOE requirement so far [130].

### 5.3. Carbon Nanotubes (CNTs) as Hydrogen Carriers

**Figure 9:**
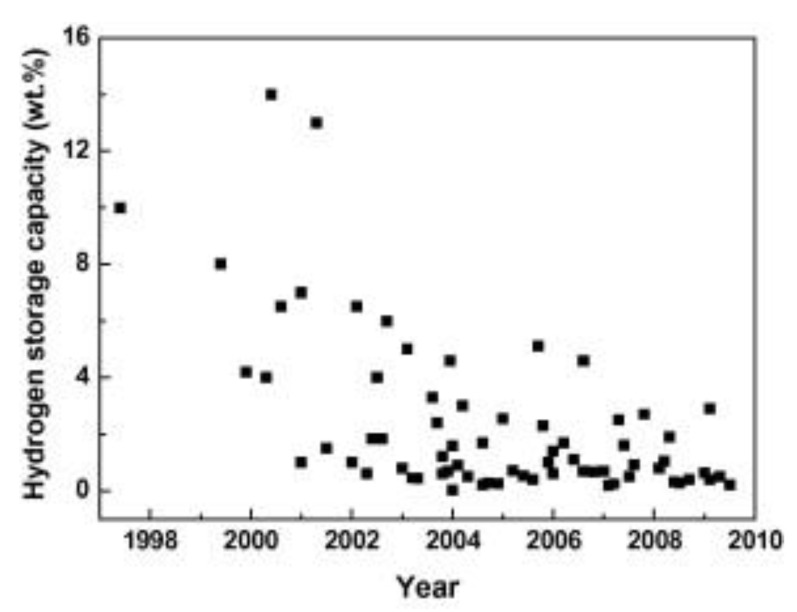
Plot of the hydrogen storage capacities of CNTs reported in the literature versus their year of publication (copyright Elsevier 2010) [136].

CNTs have been reported as one of the best carbon-based hydrogen carriers due to their high surface area and tunable nanostructure. The hydrogen molecules can adsorb in the space between the individual channels as well as the mesoporous tubes or into the 2D triangular lattices formed by the carbon atoms’ orientation (Figure 8) [131,132,133]. Over recent years, many review articles have summarized studies on the hydrogen storage ability of CNTs [131,133,134]. Like other carbon-based carriers, CNTs can adsorb a large number of hydrogen molecules at cryogenic temperatures [132,135]. Upon changing the reaction conditions from cryogenic to room temperature and lower pressure, however, the hydrogen uptake of CNTs is significantly decreased [132,136]. In an article by Liu, it was suggested that it was no longer worth investigating hydrogen uptake in pure CNTs for on-board applications due to the low hydrogen uptake of CNTs at room temperature (Figure 9) [136].

Similar to other porous materials, CNTs were hybridized with active metals, such as alkali metals, to increase their binding strength with hydrogen atoms, and these hybrids were expected to result in higher hydrogen uptake. According to Chen et al., CNTs doped with alkali metals can adsorb up to 20 wt% of H_2_ at 380 °C and 10 bar [137]. The same results were reported until 2000, when Yang et al. claimed that the results were inaccurate because of the moist environment. They demonstrated that, in the absence of moisture, the H_2_ adsorption of the same doped CNTs was only 2.5 wt% for the best composition [138].

From a glance at the review articles on CNT-based hydrogen carriers wherein different methods and materials for this purpose were compared, it becomes obvious that the hybridization of CNTs with active metals and catalysts could increase the chance of reaching the DOE target [132,133,134]. In the beginning, different treatments of CNTs were investigated to improve the surface area of pure CNTs, but the high-energy ball milling method was ultimately found to be the most efficient means of synthesizing nanocomposite hybrid materials. Metal-doped CNTs were proposed by researchers as an alternate technique for absorbing hydrogen via the spillover mechanism when CNTs were treated with various methods [139]. In this mechanism, the CNT’s surface is covered with metal nanoparticles, which are capable of dissociating hydrogen molecules. Carbon nanomaterials act as a support for the first spillover while being the source of the secondary spillover. However, recent theoretical studies have shown that the gravimetric storage limits of spillover-based materials are insufficient for on-board storage and reaching the DOE standards. Repetition of previous spillover experiments with high storage capacity also failed to confirm reproducible storage of these materials [140]. The capacity of hydrogen carriers may be increased by other mechanisms, such as multiple hydrogenated sites, according to some recent studies [141].

Over the last two decades, various composites have been discussed with respect to the various metal catalysts and to the presence/absence of additional supports. As an example, Hwang et al. studied the hydrogen storage capacities of Pt-, Ni-, and Ag-doped MWCNTs at 298 K, which were reported to be 2.9, 2.27, and 0.86 wt%, respectively [142]. In this regard, the hydrogen storage capacity of the MWCNT doped with Pd and Ni was investigated by Mehrabi et.al., where the samples were synthesized by the laser ablation and chemical reduction methods. It was found that both methods improved the hydrogen storage capacity of CNTs at high temperatures; however, the hydrogen adsorption at ambient conditions did not exceed 1 wt% [143]. Additionally, using the functionalization of the MWCNT with different nanoparticles such as SnO2 or MnO_2_ could improve the hydrogen storage capacity of these materials [144,145]. Nevertheless, none of these studies could reach the current (4.5 wt% H_2_) and the ultimate (6.5 wt% H_2_) DOE targets for mobile applications.

Recently, an article reported 10.94 wt% hydrogen uptake in TiO_2_/Fe–Ag/MWCNT hybrids based on electrochemical methods at ambient temperature [146]. Another study also presented the experimental and theoretical H_2_ storage results of the spillover of Ni nanoparticles onto N/O-rich CNTs. Regarding the combination of both physio- and chemisorption hydrogen in the same composites, a higher storage amount could be imaginable [147]. Furthermore, a more detailed investigation of these materials is needed to reach the DOE or higher targets for hydrogen fuel systems.

### 5.4. Carbon Nanofibers (CNF) as Hydrogen Carriers

CNFs consist of stacked graphene sheets in multiple arrangements, such as stacked platelets, ribbons, and herringbones (Figure 8). Typically, their length is in the order of micrometers, while their diameter is in the range of nanometers. Mechanically and electrically, CNFs are similar to CNTs, despite their different orientation of the graphene layers. Graphene sheets with a greater active surface area can provide ideal CNFs for various applications that require high surface availability. Nanofibers showed the adsorption of 16 wt% H_2_ at 120 atm and 298 K, which is as high as necessary for commercial applications [148]. However, a further study on carbon nanofibers contradicted these results; Poirier et al. reported that some CNFs only adsorbed 0.7 wt% at room temperature and 105 bar. It should be noted that the surface area of this CNF calculated by BET analysis was only 200 m^2^ g^−1^ (quite low related to normal CNFs) [149]. Another study claimed 6.5 wt% hydrogen adsorbed to CNFs prepared by the decomposition of ethylene over Fe-Ni-Cu alloys [150]. However, contradictory results for hydrogen uptake by carbon nanofibers have been presented in various studies so far [134].

The hydrogen uptake of KNO_3_-doped vapor-grown CNFs at a moderate temperature range of 303 to 873 K was investigated by thermogravimetry; 5.1 wt% hydrogen was adsorbed at 373 K and 0.1 MPa for the CNFs with diameters between 200 and 500 nm. For raw CNF, the same experiment led to the uptake of hydrogen gas in the amount of 0.34 wt%, indicating that doping with KNO_3_ did have a remarkable effect on CNF–H_2_ interactions [151]. In addition to the modification of these materials, there are other ways to improve the hydrogen adsorption of CNFs. The treatment of the CNFs with KOH enhanced the specific surface area of nanofibers; however, this activation method can destroy the structure of the fibers under severe conditions [152]. Yadav et al. have produced an adsorbent based on nickel and carbon nanofibers for hydrogen storage. The BET surface area of the samples was increased using steam activation at 600, 750, and 900 °C. The H_2_ storage capacities of Ni-CNF were compared at various temperatures (77, 273, 298 K). A Ni-carbon microfiber with a specific surface area of 774 m^2^ g^−1^ adsorbed the highest number of hydrogen molecules (0.75 wt%) at room temperature and 50 bar [153]. Li–fluorine-co-doped carbon nanofibers showed a hydrogen storage capacity of 2.4 wt% at 0 °C and 10 MPa. As a result of the large electronegativity difference between fluorine and lithium, hydrogen molecules could be stabilized more effectively in doped CNFs compared to pure CNFs [154].

A recent article by Hwang et al. investigated the effects of surface modification and hollow porous structures on the hydrogen capacity of CNFs. The highly porous carbon fibers derived from chemical activation have a specific surface area of 3058 m^2^ g^−1^, which is significantly higher than that of other CNFs reported before. Additionally, 5.14 wt% hydrogen was adsorbed to the surface of activated CNF at 77 K and 10 MPa, while the number increased to 5.43 wt% in the presence of Pd nanoparticles [155].

So far, carbon materials have been found to be capable of adsorbing a limited amount of H_2_ under ambient conditions, which is not convenient for mobile applications. Table 2 presents the highest level of hydrogen adsorption reported by carbon family. It is obvious that some of these results are no longer permissible and could not be reproduced by new experiments. Regarding these results, hydrogen storage in carbon materials is still an open topic, and many further investigations are needed to understand the effective factors in hydrogen uptake.

## 6. Summary

**Figure 10:**
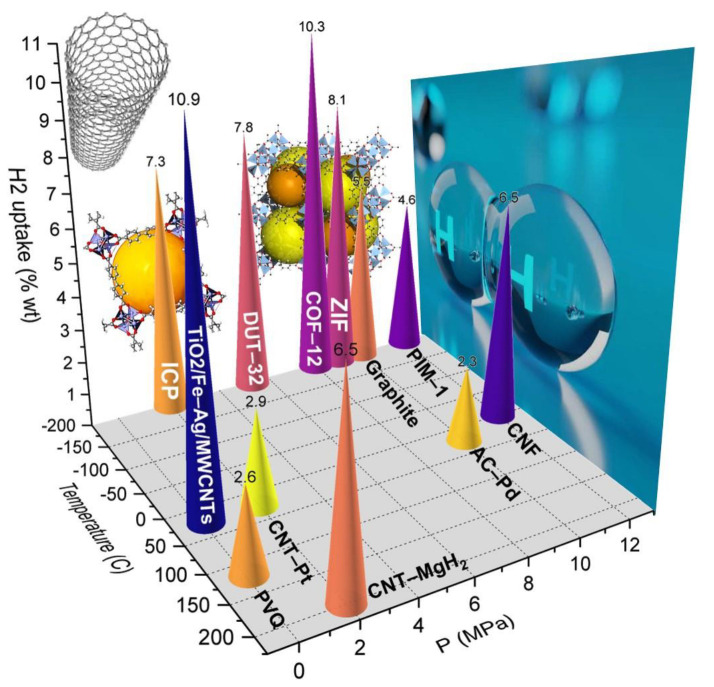
An overview of polymer-based hydrogen storage carriers.

In order to understand and design improved hydrogen carriers, thermodynamic parameters, such as hydrogenation and dehydrogenation enthalpies, temperature, pressure, and kinetic properties, such as activation energy, are necessary. To improve these properties in adsorption-based carriers, many strategies, such as particle size control, surface area improvement, porosity adjustment, synthesis of uniform structure with given materials, etc., have been studied. However, nanoporous materials prepared from polymers still need to be studied and improved, since these existing systems do not meet the DOE requirements yet (Figure 10). New investigations are also needed to address the lack of polymers with the highest hydrogen capacity in absorption-based carriers. In the future, hydrogen-rich polymers might serve as effective hydrogen storage materials.

It should be mentioned that there is still a large gap between polymer-based hydrogen storage capacity and the DOE targets suggested for 2025 and the ultimate one, which is 6.5 wt% with an operating temperature in the range of −40 to 60 °C. The main hindrance to suitable hydrogen density materials for practical applications are hydrogenation/dehydrogenation temperature, operation pressure, reversibility, and kinetics of operation. It has been suggested that machine learning tools could be used to analyze all data provided so far to show the future of this field. While analyzing these data, the requirement of descriptor parameters needs to be determined for hydrogen storage at ambient temperature and pressure.

Future polymer-based hydrogen storage carriers can be improved by reducing the hydrogen desorption temperature and increasing their capacity: (i) nanosizing the additives materials to maximize their catalytic effects for nanoporous systems; (ii) developing new effective nano-frameworks with higher surface areas and porosity densities by using the MOF or COF systems; (iii) investigating the hydrogen absorption of new and functional polymers.

## 7. Conclusions

While climate change has both natural and anthropogenic components, global warming caused by greenhouse gases, especially CO_2_, is the main cause of anthropogenic climate change. According to the current situation, fossil fuels are the biggest source of CO_2_ emissions and climate changes and have been replaced by clean energies. The economy, energy usage, environment, and climate are all interconnected, but “securing energy and the environment at the lowest cost” is the reason for hydrogen storage efforts. Hydrogen as a practical energy vector for storage is proposed as a renewable energy source for stationary and mobile applications. Several hydrogen storage systems have been presented, considering their application in the mobility industry. Compressed hydrogen cylinders are bulky and expensive, but they are the current choice of industry for practical reasons, even if they do not achieve the DOE system target. Over the last century, the concept of chemical hydrogen storage through adsorption and absorption, especially with polymerbased carriers, was brought to light. In this review, both the advantages and drawbacks of the polymeric hydrogen carriers were separately discussed. These included complicated thermal management systems, costly catalysts, and operating conditions. The purity of hydrogen gas released from polymer-based hydrogen carriers and the rechargeability of these systems should be studied. However, none of the polymer-based hydrogen carriers could reach the DOE target of 6.5 wt% with an operating temperature in the range of −40 to 60 °C without considering final costs.

## Figures and Tables

**Table 1:** Hydrogen capacity of the selected polymers. These last three rows represent the polymers suggested by the authors (references [50,51,52] relate to the corresponding low-molecular-weight component).

Polymer	Hydrogenated Form	Molecule Weight of Monomer (g.mol^−1^)	Hydrogen Capacity (%wt)	Hydrogenation Condition	Ref.
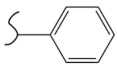	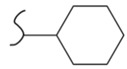	104	4.80	473 K, Ni catalyst	[39]
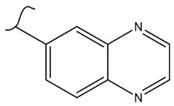	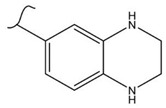	160	2.5	333 K, Ir catalyst 5 h	[37]
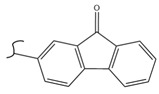	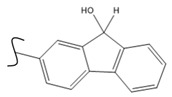	208	0.96	373 K, Ir catalyst 5 h	[44]
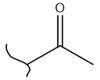	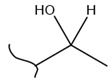	72	2.77	453 K, Ir catalyst 1.5 h	[42]
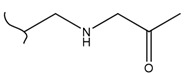	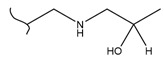	115	1.73	368 K, Ir catalyst 8 h	[48]
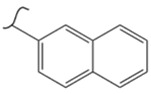	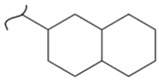	164	6.1	393 K, Ni catalyst 20 bar	[50]
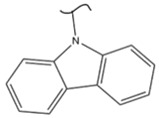	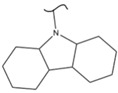	205	5.85	453 K, Pt or Pd catalyst 20–100 bar	[51]
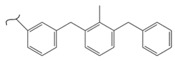	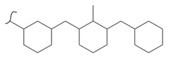	319	5.64	593 K, Pt catalyst >10 bar	[52]

**Table 2:** Experimentally measured hydrogen storage capacity of different carbon-based carriers.

Materials Group	H_2_ Gravimetric Capacity (%wt)	Process Condition	Carrier Properties	Ref.
AC	0.70	303 K, 10 MPa	SSA: 3306 m^2^ g^−1^ Pore volume: 1.2 cm^3^ g^−1^	[156]
AC (KOH-treated)-Oxygen-rich	8.70	77 K, 30 MPa	SSA: 3800 m^2^ g^−1^ Pore volume: 1.8 cm^3^ g^−1^	[157]
SWNH	0.50	303 K, 10 MPa	SSA: 910 m^2^ g^−1^ Pore volume: 0.31 cm^3^ g^−1^	[156]
AC (KOH-treated)	6.60	77 K, 8 MPa	SSA: 3451 m^2^ g^−1^ Pore volume: 1.05 cm^3^ g^−1^	[123]
Platinum-doped AC/MOF5	2.30	298 K, 100 MPa	SSA: 730 m^2^ g^−1^ Pore volume: 0.303 cm^3^ g^−1^	[125]
CNT (Li-doped)	20.00	678 K, 10 MPa	Energy density: 0.133 KWh/Kg	[137]
GNF	58.37	298 K, 12 MPa		[148]
SWCNT	4.20	298 K, 10 MPa		[158]
SWCNTs (50% purity)	1.70	290 K, 12.04 MPa	SSA: 229 m^2^ g^−1^	[136]
Pt-alloy CNT	2.90	298 K, 3.2 MPa	Diameter: 10–40 nm	[142]
Ni-alloy CNT	2.27	298 K, 3.2 MPa	Diameter: 10–40 nm	[142]
Ag-alloy CNT	0.86	298 K, 3.2 MPa	Diameter: 10–40 nm	[142]
TiO2/Fe–Ag/MWCNT	10.94	298 K, 0.1 MPa		[146]
MgH2-CNT	6.50	473 K, 2 MPa		[159]
KOH-MWCNT	4.47	823 K		[160]
Treated-CNF	6.50	273 K, 12 MPa	SSA: 51 m^2^ g^−1^ Fiber Diameter: 50–260 nm	[150]
KNO3-doped CNF	5.10	373 K, 0.1 MPa	Diameter: 200–500 nm	[151]
Pd-doped Activated CNF	5.43	77 K, 10 MPa	SSA: 3058 m^2^ g^−1^	[155]

AC: activated carbon, SWNH: single-walled carbon nano-horns, CNT: carbon nanotube, SWCNT: single-wall carbon nanotube, MWCNT: multi-wall carbon nanotube, CNF: carbon nanofiber.

## Data Availability

The data presented in this study are available on request from the corresponding author.
